# Evolutionary Changes in Primate Glutamate Dehydrogenases 1 and 2 Influence the Protein Regulation by Ligands, Targeting and Posttranslational Modifications

**DOI:** 10.3390/ijms25084341

**Published:** 2024-04-14

**Authors:** Yulia A. Aleshina, Vasily A. Aleshin

**Affiliations:** 1 Martsinovsky Institute of Medical Parasitology, Tropical and Vector Borne Diseases, Sechenov First Moscow State Medical University, 119435 Moscow, Russia; 2 Faculty of Bioengineering and Bioinformatics, Lomonosov Moscow State University, 119234 Moscow, Russia; 3 Belozersky Institute of Physicochemical Biology, Lomonosov Moscow State University, 119234 Moscow, Russia; 4 Department of Biochemistry, Sechenov First Moscow State Medical University, 119048 Moscow, Russia

**Keywords:** glutamate dehydrogenase, *GLUD2*, human evolution, allosteric regulation, posttranslational modifications, mitochondrial targeting sequence, mitochondrial signaling factors, hyperinsulinism/hyperammonemia, Parkinson’s disease

## Abstract

There are two paralogs of glutamate dehydrogenase (GDH) in humans encoded by the *GLUD1* and *GLUD2* genes as a result of a recent retroposition during the evolution of primates. The two human GDHs possess significantly different regulation by allosteric ligands, which is not fully characterized at the structural level. Recent advances in identification of the GDH ligand binding sites provide a deeper perspective on the significance of the accumulated substitutions within the two GDH paralogs. In this review, we describe the evolution of GLUD1 and GLUD2 after the duplication event in primates using the accumulated sequencing and structural data. A new gibbon *GLUD2* sequence questions the indispensability of ancestral R496S and G509A mutations for GLUD2 irresponsiveness to GTP, providing an alternative with potentially similar regulatory features. The data of both *GLUD1* and *GLUD2* evolution not only confirm substitutions enhancing GLUD2 mitochondrial targeting, but also reveal a conserved mutation in ape GLUD1 mitochondrial targeting sequence that likely reduces its transport to mitochondria. Moreover, the information of GDH interactors, posttranslational modification and subcellular localization are provided for better understanding of the GDH mutations. Medically significant point mutations causing deregulation of GDH are considered from the structural and regulatory point of view.

## 1. Introduction

Mammalian glutamate dehydrogenase (GDH) is an NAD(P)-dependent GDH (EC: 1.4.1.3) catalyzing reversible oxidation of L-glutamate to 2-oxoglutarate and ammonia. Its function is indispensable for the metabolism of the neurotransmitter glutamate in the brain [1]. The role of GDH as an enzyme controlling anaplerotic flux fulfilling the TCA cycle is also hard to overestimate. As an ammonia-producing/assimilating enzyme, GDH is relevant for kidney function [2] and for the metabolism and progression of some tumors [3]. Thus, GDH is an essential enzyme linking carbon and nitrogen metabolism, which is relevant for all tissues.

GDH is found in all living organisms [4], with two different structural subfamilies of small and large GDH identified [5,6]. All mammalian GDHs belong to the subfamily of small GDH with subunits of ~55 kDa. These hexameric enzymes are organized as a dimer of trimers [4], which may form supramolecular filamentous structures of higher molecular weight [7,8]. Mammalian GDH was one of the first discovered allosteric enzymes [9]. Still, the available structural data do not allow complete characterization of GDH regulation by its ligands, and the accumulation of new data fills the gap, providing further understanding of GDH allosteric regulation [10].

Although GDH is an essential enzyme for the human body, particularly necessary for brain function, its role is far from being fully understood. This is partially due to the GDH gene (*GLUD1*) duplication in humans [11]. The second intronless *GLUD2* gene is specific to hominoids/apes (gibbons and great apes) and emerged due to a retroposition less than 23 million years ago [12]. The *GLUD2* gene is located on the X chromosome, while the *GLUD1* gene is located on chromosome 10. A burst of retropositions in primates began ∼40–50 million years ago, generating a large number of copies of genes [13]. In addition to the protein-coding genes *GLUD1* and *GLUD2*, five GDH pseudogenes (*GLUD1P2*-*GLUD1P6*) are present in humans [14]. At least three of these pseudogenes are RNA-coding [15]. The presence of accumulated pseudogenes illustrates the evolutionary process of GDH duplication and elimination/modification of unnecessary copies by spontaneous mutations. In contrast to the five *GLUD1* pseudogenes, the *GLUD2* gene is protein-coding with retained enzymatic activity. Its appearance and evolution into the current form approximately coincide with the period of increase in primate brain size and complexity [12].

The fact that the novel *GLUD2* gene has not become a pseudogene means it is beneficial to its carriers. Positive selection has been suggested to have led to accelerated protein evolution after GDH duplication, while new functional variants in individual ape lineages were maintained by purifying selection [12]. The absence of *GLUD1* amino acid substitutions in the ”mature GDH”-coding region has been reported, suggesting sharp purifying selection for this gene. However, the mitochondrial targeting sequence (MTS) of GDH, which is predominantly 53 amino acids long in primates, is neglected in most studies [12,16], although alterations in this region can affect the localization and function of the enzyme [17,18]. Focused analysis of *GLUD2* MTS reveals specific mutations in this region, likely causing more efficient mitochondrial targeting [19]. Still, little attention has been given to *GLUD1* sequences. Since the characterization of evolution of GDH sequences by Burki and Kaessmann [12] and Rosso et al. [19], new genomic data have been accumulated; thus, here we aimed at an independent revision of the available sequences. These data are compared and studied in the context of GDH evolution in primates. Novel structural data on mammalian GDH are applied for the review of GDH evolution and available data about genetic variants of *GLUD1* and *GLUD2* from patients.

## 2. Multiple Ligand Binding Sites of the Mammalian GDH

**Figure 1:**
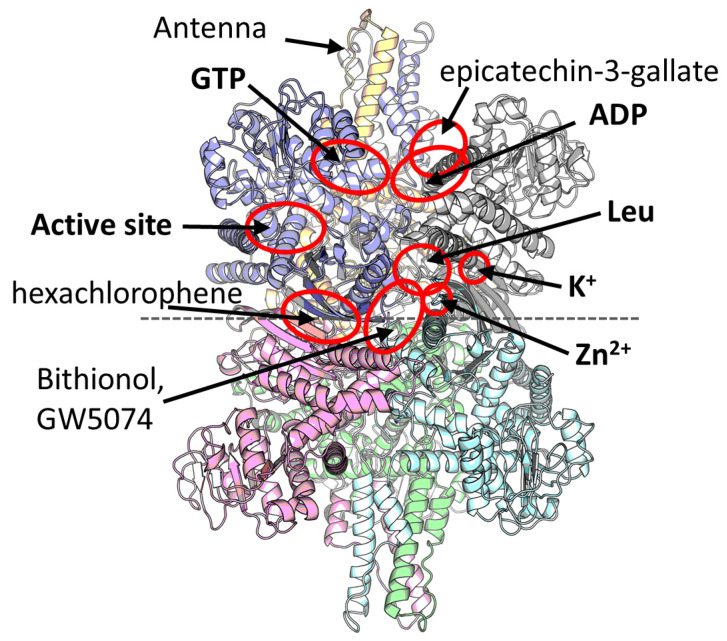
A model of the mammalian GDH and its ligand binding sites. Six GDH subunits are shown in different colors. Two trimers of the hexamer are indicated by a dashed horizontal line. Two antenna regions are built by three subunits each and located at the top and the bottom of the hexamer. The model is represented by a GDH structure with Leu, ADP, and K^+^ (PDB ID: 8AR7 [10]) in an open conformation. Overall composition of the closed conformation is the same; however, different ligands (often either activators or inhibitors) stabilize specifically the open (e.g., ADP, Leu) or closed (e.g., GTP, Zn^2+^) conformation. The composition and structure of GLUD2 is similar to that of GLUD1 [39]. Red ovals indicate the active site and experimentally shown allosteric sites, of which natural physiologically relevant regulators are written in bold.

Mammalian GDH was one of the first discovered allosteric enzymes, with the activatory effect of ADP and inhibitory effect of GTP being known for more than 60 years [9]. However, the GDH structures comprising these ligands were characterized nearly 40 years later, revealing two separate allosteric inhibitory (GTP) and activatory (ADP) binding sites (Figure 1) [8,20]. ATP is supposed to bind primarily to the GTP binding site [21]. Additionally, leucine [22], diethylstilbestrol/estrogens [9], palmitoyl-CoA [23] and Zn^2+^ [24] have also been known as the natural regulators of the mammalian GDH. Of these natural regulators, localization of a Zn^2+^ binding site [25] and, only recently, a leucine binding site found together with a novel K^+^ ion site [10] have been identified (Figure 1). Surprisingly, all these ligands possess separate allosteric regulatory sites, and even more binding sites have been identified with the help of crystallization screenings. That is, a binding site within the central GDH cavity has been found upon crystallization with hexachlorophene and, also, another site was found upon crystallization of GW5074 or bithionol [26]. Additionally, an epicatechin-3-gallate binding site was localized [27] that partially overlaps with the ADP site (Figure 1). Thus, eight different ligand binding sites other than the active site are available in mammalian GDH (Figure 1). However, despite more than 20 structures of mammalian GDH available in PDB, the binding sites of even natural regulators such as palmitoyl-CoA [23], estrogens/diethylstilbestrol [9,28,29], thiamine or its derivatives [10,30], or haloperidol, perphenazine [29] and many other ligands [31,32] remain to be identified.

In addition to the regulatory sites themselves, mammalian GDH structures possess a special antenna region made of six α-helices from three different GDH subunits (Figure 1). Within each subunit, this region extends from the NAD domain and undergoes conformational changes between the opened and closed conformations of the enzyme active site. The antenna region is unique for the animal GDH, first appearing in the ciliates [33]. The region serves to facilitate the intersubunit communication and allosteric regulation of animal GDH [20]. Replacement of the human GDH antenna with a short loop found in bacterial GDH from *Clostridium symbiosum* caused loss of sensitivity to ADP, GTP, and palmitoyl-CoA [33]. However, complete removal of the antenna makes the human GDH extremely amenable to ADP activation, although it lowers basal activity [34]. These and other published experiments on the antenna region highlight the role of the antenna for the catalytic turnover rate of the enzyme and its allosteric regulation [33,34]. Nevertheless, the GDHs from bacteria and fungi possessing no antenna may also be allosterically regulated [35,36,37,38].

Studies of the “mature” recombinant forms of the two human GDHs have revealed that the GLUD2 protein is more thermolabile and its basal activity is about ten times lower compared to GLUD1 [40]. However, GLUD2 is practically insensitive to physiological GTP concentrations and its activation by ADP and/or leucine is more than 10-fold higher than that of GLUD1 [40,41]. These studies also have demonstrated slight inhibition of recombinant GLUD1 but not GLUD2 with millimolar Mg^2+^ concentrations [41]. However, in the presence of ADP, no effect of Mg^2+^ or Ca^2+^ has been detected on pure GDH, while Mn^2+^ inhibited ADP-activated GLUD2 stronger than GLUD1 [42]. The effect of impurities present in cell extracts on the GDH inhibition by cations has been noticed [42]. GDH inhibition by millimolar Mg^2+^ and Mn^2+^ could resemble the regulation of the enzyme by its physiological inhibitor Zn^2+^ at micromolar concentrations [25], since Zn^2+^ concentration range *in vivo* has been estimated as 25–100 μM [43]. Of note, X-linked GLUD2 turned out to be significantly more sensitive to estrogens/diethylstilbestrol but not to testosterone compared to GLUD1 [28,29]. A higher sensitivity of GLUD2 to palmitoyl-CoA has been reported [44]. Thus, studies of the two human GDH isoforms have revealed that hominoid-specific GLUD2 possesses lower basal activity, which, however, is insensitive to GTP inhibition and is more responsive to activation by ADP and leucine and inhibition by estrogens and palmitoyl-CoA. The allosteric regulation of these two GDH forms differs so much that it could promote the positive selection of the *GLUD2* gene soon after its retroposition [12,19,45].

The evolution and properties of the hominoid-specific GDH has driven high attention since the discovery of the second GDH gene in humans, *GLUD2* [11,16,19,40]. Positive selection of this gene suggests that some amino acid substitutions within this protein were beneficial for its function [12]. Mutations of the amino acid residues localized within GDH regulatory sites would likely influence the effect of the ligands involved. To evaluate the potential effect of mutations on the GDH regulation, we summarize the evolutionary changes in the *GLUD1* and *GLUD2* genes of apes and put them together with all the accumulated structural data on mammalian GDH ligand sites.

## 3. Evolution of Glutamate Dehydrogenases in Hominoids

As evaluated before, duplication of the GDH gene occurred 18–23 million years ago, after the Old World monkey–hominoid split, but before the separation of the gibbon lineage (family Hylobatidae) from that of humans and great apes [12]. The study included African green monkey (*Chlorocebus sabaeus*) as the only animal representing Old World monkeys. Gibbons were represented by *Hylobates lar*; great apes were represented by orangutan—*Pongo pygmaeus*, chimpanzee—*Pan troglodytes*, and gorilla—*Gorilla gorilla gorilla*. To date, the Uniprot database also includes sequences annotated as *GLUD2* from *Pongo abelii*, *Nomascus leucogenys*, *Rhinopithecus roxellana*, *Cercocebus atys* and *Mandrillus leucophaeus*, with the last three species being representatives of the Old World monkeys. Such a contradiction with the previous study can be explained by the wrong annotation of the sequences from *Rhinopithecus roxellana*, *Cercocebus atys* and *Mandrillus leucophaeus*. Careful comparison of the sequences and their annotations showed the absence of the GDH gene on the X chromosome of these species. Notably, no sequences of *GLUD1* from gibbon species are currently available in Uniprot [46]. Theoretically, the original gene could become a pseudogene due to spontaneous mutations after the duplication, resulting in the absence of *GLUD1* in gibbons. Such an event is unlikely, although possible. Thus, analysis of new available GDH data from more primate species than evaluated before is clearly needed.

### 3.1. GDH Duplication in Primates

**Figure 2:**
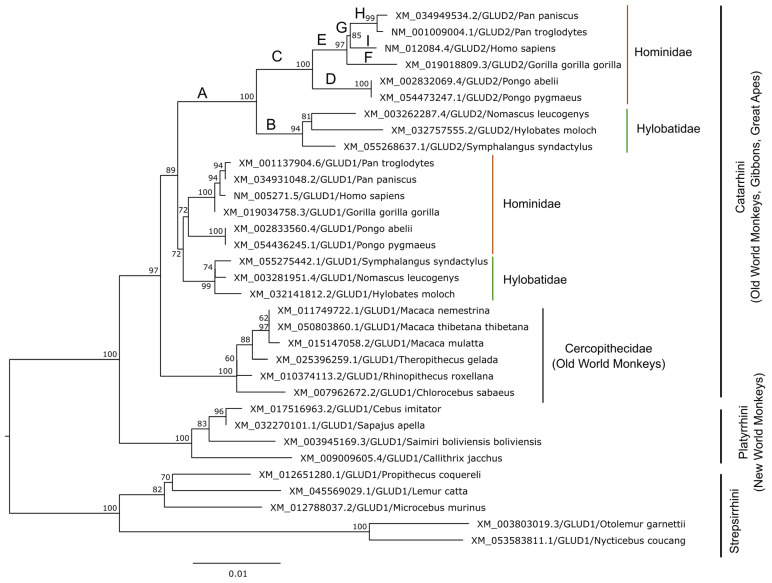
Maximum likelihood tree of *GLUD1* and *GLUD2* coding sequences (with MTS) available for primates in RefSeq database [48]. The tree was inferred using the IQ-TREE v2.0.6 program [49] incorporating the best-fit nucleotide substitution model (K2P + R2) [50]. The number of ultrafast bootstrap replicates was 10,000 [51]. The ultrafast bootstrap supports (%) are indicated at the tree nodes. The GLUD2 clades (A–I) are marked starting from the common GLUD2 ancestor (A) in the order of division of particular evolutionary branches. The substitutions that occurred in sequences of these clades are further discussed in Section 3.3.

As of March 2024, there were *GLUD1* orthologs from 25 primate species in the NCBI Orthologs database [47]. Due to the presence of an ambiguous nucleotide in the mRNA sequence of GLUD1 from *Aotus nancymaae* (parvorder Platyrrhini, XM_012472623.2), this sequence was omitted for the subsequent analysis. Of the remaining 24 species, 9 possess a GLUD2 sequence. The phylogenetic tree inferred using *GLUD1* and *GLUD2* coding sequences available for primates in the RefSeq database [48] supports the GDH duplication in the common ancestor of hominoids (gibbons and great apes including humans), who all have two copies of GDH, while other primates have only *GLUD1* (Figure 2).

There is a common opinion that human *GLUD1* has not been changing after duplication [16]. The phylogenetic analysis shows that, indeed, the tree branches in the ape *GLUD1* clade are shorter compared to those of the *GLUD2* clade and suggests that *GLUD2* has accumulated more mutations (Figure 2). The *GLUD1* coding sequences from apes accumulated from 10 to 13 nucleotide substitutions after the duplication event, and the vast majority of these mutations are synonymous.

### 3.2. Specific Properties and Expression of GLUD2

Since *GLUD2* remains a protein-coding gene, its acquirement provides a new beneficial feature to its carriers, or possibly even several ones, due to the multiple functions of GDH. Here, we will summarize such potentially relevant specific features of *GLUD2* and put them into the context of its functions.

One of the main novel features of *GLUD2* is its intronless structure. This is a very important aspect for mRNA nuclear export and transcription, which normally require splice factors [52]. Secondly, *GLUD2* is localized on the X chromosome, resulting in (1) its copy number sex difference and also likely (2) its silencing during spermatogenesis due to the sex chromosome inactivation [53]. As a result, it enables a complex regulation of *GLUD2* expression. Many intronless genes are predominantly expressed in the testes and nervous tissue [52]. –So is *GLUD2* [11].

*GLUD2* mRNA was first detected in the brain and testes but not in liver [11], which slightly contradicts the expression databases FANTOM5, Genotype-Tissue Expression project and Human Protein Atlas, where a substantial liver expression has been detected [15,54,55]. Analysis of the protein product levels has revealed high expression of GLUD2 in Sertoli cells. No protein product is detected in liver, but GLUD2 is identified in astrocytes [56]. GLUD2 protein is also found in renal proximal tubules, placenta and adrenals [2,57]. These data are in good agreement with the single-cell RNA expression data added to the Human Protein Atlas [55,58]. According to them, *GLUD2* RNA is most expressed in Sertoli cells, then in cytotrophoblasts, distal and proximal tubular cells, syncytiotrophoblasts, astrocytes and also in many other cells with slightly lower expression, including excitatory neurons and a subgroup of hepatocytes.

Comparison of the *GLUD2* expression profile in different tissues [15,54,55] with the data on protein levels in these tissues [2,56,57] indicates a suppressed expression of the intronless RNA, which is highly likely not active in liver [52]. Alternatively, translation repression could take place. Indeed, *GLUD2* repression by miR-27a has been proposed [59,60]. Additionally, there are GDH pseudogenes in the human genome [14], and at least three of them are RNA-coding [15]. Such RNA could also be involved in the regulation of expression of *GLUD1* and *GLUD2*.

The function of *GLUD2* in Sertoli cells or cytotrophoblasts has not been studied extensively. However, it can be proposed that the main role of the GLUD2 protein in these cells is its GTP-independent glutamate oxidation. One of the functions of Sertoli cells is lactate production, which is the main energy source for the germ cells [61]. These cells use a lot of glucose and convert it into lactate. In addition, glutamine is used to support the oxidative metabolism and fuel the TCA cycle with the help of glutaminase and glutamate dehydrogenase [61]. The cytotrophoblasts also use glutamate for oxidative metabolism. Interestingly, labelled glutamate has a higher impact for ^14^CO_2_ production than glutamine, and GDH inhibition significantly downregulates ^14^CO_2_ production [62]. Thus, both cell types with the highest *GLUD2* expression—the Sertoli cells and cytotrophoblasts—likely use the GTP-independent form of GDH for their oxidative metabolism, supporting high ATP and GTP production in mitochondria.

Noteworthily, human *GLUD2* could substitute testicular *Bb8* GDH of *Drosophila melanogaster* better than human *GLUD1* or *Drosophila* housekeeping *Gdh* [63]. A role for the difference in GDH regulation can be proposed. In addition, Vedelek et al. propose that the *Bb8* protein could form filamentous and lamellar structures similar to mammalian glutamate dehydrogenases [7,8]. Then, the ability of *GLUD2* to substitute for the *Bb8* GDH suggests its potential role in the formation of such filamentous structures when a sufficient local concentration can be achieved.

*GLUD2*’s function in the brain has driven more attention. It has been noted that astrocytes produce lactate, which fuels learning-induced mRNA translation in neurons [64]. This function of astrocytes is likely similar to that of Sertoli cells. Glioma studies have discovered that *GLUD2* expression promotes cell growth and metabolite flux to lipids in IDH1^R132H^ glioma progenitors in mice [65]. That is, R132H mutation, which disrupts normal function of isocitrate dehydrogenase IDH1, negatively affects the growth of glioma progenitor cells, but genetically induced expression of human *GLUD2* but not *GLUD1* rescues the cells. Glutamate could support the growth of glioma progenitors irrespective of IDH1 mutation status. It has been suggested that specialization of the human neocortex for high glutamate neurotransmitter flux creates a metabolic niche conducive to the growth of IDH1 mutant tumors [65]. Indeed, knockdown of the *GLUD2* gene indicates that the gene is required for the glutamate-dependent growth of glioma [66]. The IDH1 transfection experiments also suggest that glioma cellular growth is downregulated by the lack of 2-oxoglutarate rather than accumulation of oncometabolite 2-hydroxyglutarate [66]. However, overexpression of *GLUD2* in human IDH1^wt^ glioblastoma T98G and U118 cells inhibits cell growth [67]. *GLUD2* expression increases the capacity for glutamate uptake and oxidation, particularly during increased workload and aglycemia, in cultured astrocytes of *GLUD2*-expressing transgenic mice [68]. It also increases the utilization of branched-chain amino acids during aglycemia, causing a general decrease in the oxidative metabolism of glucose. Thus, *GLUD2* could provide human astrocytes with an ability to spare glucose in case of its shortage. Notably, the introduction of *GLUD2* does not affect glutamate levels in mice. Instead, *GLUD2*’s metabolic effects center on the TCA cycle and the carbon flux [69].

In glutamatergic neurons, expression of *GLUD2* may strengthen excitatory glutamate neurotransmission [70]. Targeted overexpression of *GLUD1* in mouse cortical neurons (via a neuronal-specific promoter), which partially resembles the presence of GTP-resistant *GLUD2*, increases presynaptic glutamate release [71].

### 3.3. Structural Analysis of Mutation Sites Occuring upon Evolution of “Mature” GLUD2

**Figure 3:**
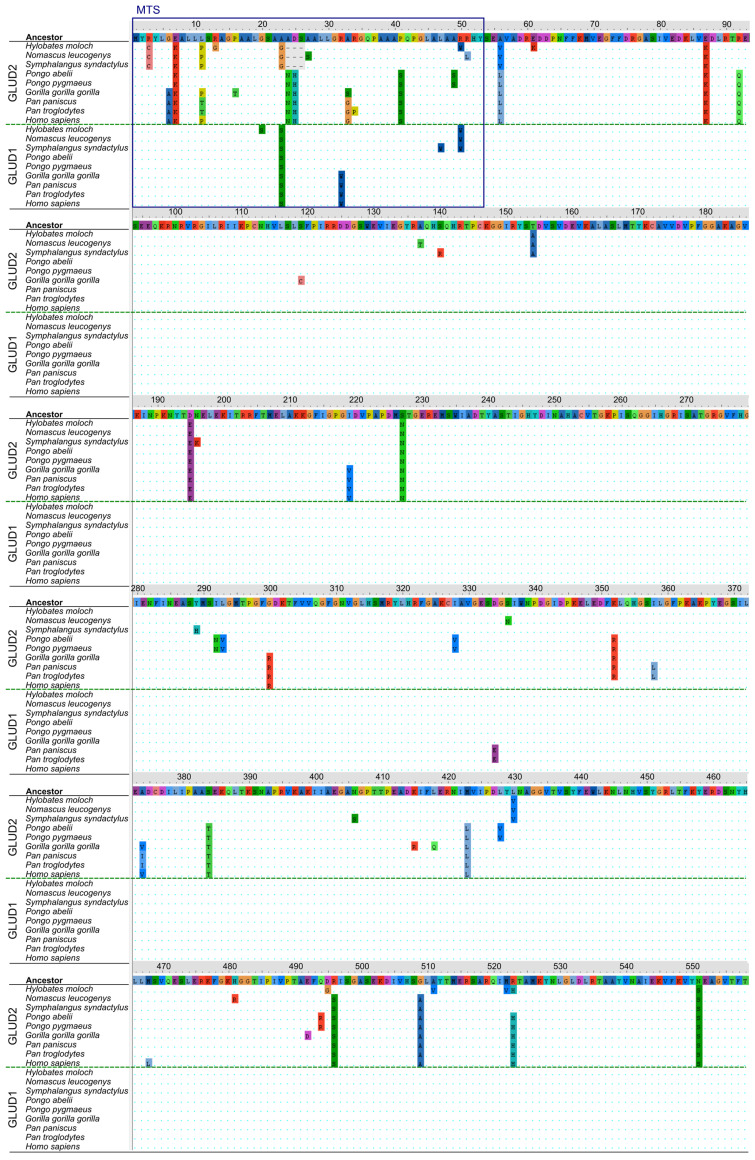
Multiple sequence alignment for translated *GLUD1* and *GLUD2* coding sequences from apes and their ancestor. The ancestral sequence was inferred using the maximum likelihood method [74] under the Kimura 2-parameter model [75] implemented in MEGA11 [76]. The analysis involved coding sequences of *GLUD1* and *GLUD2* from apes. The IDs of the sequences are indicated in Figure 2. Positions 1–53 correspond to the MTS (marked with blue rectangle). *GLUD2* and *GLUD1* are separated by a dashed green line. Dots indicate amino acids that are identical to the ancestral sequence. The alignment was visualized in AliView v1.28 software [77].

Changes in “mature” GLUD2 have been extensively studied, based on the sequences of five species (human, orangutan, chimpanzee, gorilla and gibbon) obtained in the first study devoted to the origin and evolution of *GLUD2* [12,16]. Meanwhile, little attention has been given to *GLUD1* of apes. However, emergence of the *GLUD2* gene, partially substituting for the function of *GLUD1*, could affect the evolution of *GLUD1* too. Also, sequence comparisons of GDH are most commonly focused on the “mature” forms [12], which is even reflected in the numeration of GDH protein residues, often starting with Ser (typically Ser54) after MTS numbered as the first amino acid instead of Met1 [12,16]. However, such comparisons do not take into account the potential role of extramitochondrial GDH [72,73]. To fill this gap, we compared the mutations in all GLUD1 and GLUD2 full protein sequences of apes available in RefSeq (Figure 3). To trace the changes along the evolutionary history of GLUD1 and GLUD2, we reconstructed the ancestral sequences at nodes of the phylogenetic tree (Figure 2) using the maximum likelihood method [74]. As our study includes an analysis of the MTS, the numeration of the amino acids in the protein starts with Met1. The numeration of amino acids in most sequences is identical to the human GLUD1/GLUD2 or the ancestral GDH sequence, with the exception of GLUD2 of gibbons (Figure 3). A common numbering corresponding to the human/ancestral GDH sequence is used further to exclude confusion; however, a three-residues-shorter MTS of the gibbon GLUD2 sequences should be taken into account.

First, we discuss the “mature” part (positions 54-558) of GDH, putting the observed mutations in the structural context. The protein sequence of “mature” GLUD1 is indeed preserved in most organisms possessing GLUD2, which corresponds to the observations in previous studies [12]. The only exception is a non-synonymous mutation D334E in both common chimpanzee (*Pan troglodytes*) and pygmy chimpanzee (bonobo) (*Pan paniscus*) (Figure 3). The mutated residue is localized within the NADPH binding site, 4.2 Å from the 2′-phosphate group of the ligand (PDB ID: 3ETE [26], Supplementary Figure S1). While D334 itself is not involved in NADPH binding, the S333 residue forms a hydrogen bond with 2′-phosphate of NADPH, and the D334E mutation could influence repulsion between the negatively charged residue and the substrate (Supplementary Figure S1). Thus, it can be proposed that the D334E mutation of GLUD1 in chimpanzees (Figure 3) could improve NADP(H) binding to the enzyme.

While the GLUD1 protein sequence is mostly conserved, missense mutations can be found in 39 positions of the “mature” GLUD2 sequence (Figure 3). A summary of the localization of non-synonymous mutations of GLUD2 within the GDH structure is provided in Table 1. The emergence or removal of principal features of GDH upon the mutations are also summarized based on the analyzed GDH structures with available ligands (Figure 1) and published data.

Our analysis reveals twelve new mutation sites in the gibbon (Hylobatidae) branch, of which one mutation, R523H, has been previously described only for the common ancestor of great apes and humans [12]. This substitution occurred independently in *Hylobates moloch* (originally His520 residue number due to a shorter MTS in gibbons) after the division of gibbon ancestors from the other groups (Table 1, Figure 2). Simultaneously, two reverse mutations—S496R and A509G (originally Arg493 and Gly506)—are identified in *Hylobates moloch*, resulting in the same residues in these positions as in the common ancestor or in GLUD1 (Arg496 and Gly509, respectively) (Table 1, Figure 3). Other *Hylobates moloch*-specific mutations are D495G, A511V and M522V (Table 1, Figure 3). The substitution E61K, observed before, has proven to be specific to the *Hylobates* genus (Table 1, [12]). Four mutations, namely, S140R, N196K, Y289H and N406S, are specific to *Symphalangus syndactylus*, and three mutations, namely, A137T, S336N and H481R, are found only in *Nomascus leucogenys* (Table 1).

Identification of the reverse mutations S496R and A509G in *Hylobates moloch* is highly important, since mutations of these two sites, thought to be common for all GLUD2 until now, have been suggested as the ones most significantly affecting GDH regulation and stability [45,82,83,91]. However, it is admitted that the two mutations alone do not cover all the characteristics of GLUD2 [45]. Substitution R496S is known to be sufficient to almost impair both the catalytic and the allosteric functions of human GLUD1 [81]. Particularly, GTP inhibition is disrupted. However, double mutant R496S-G509A is more stable and potent to allosteric activation by ADP [45,81]. The irresponsiveness to GTP inhibition is considered to be the major allosteric property of GLUD2, enabling the GDH functioning at high cellular GTP (ATP) concentrations. The discovery of GLUD2 possessing Arg496 and Gly509 as a result of reverse mutations indicates that either the main feature of GLUD2 is not its irresponsiveness to GTP inhibition, which contradicts our current understanding of the main GLUD2 difference from GLUD1 [16], or these mutations are not mandatory for this property. The intronless structure, localization on the X chromosome and different tissue/cellular expression profile may be the alternative GLUD2 features that contribute to its unique role.

However, the importance of the R496S and G509A substitutions in the GLUD2 common ancestor should not be underestimated. As we can see from the *Hylobates moloch* sequence, its reversal to Arg496 and Gly509 (originally Arg493 and Gly506) is accompanied by D495G, A511V, M522V and R523H mutations (Table 1). All these mutations are localized either in the antenna region or in the pivot helix of GDH (Table 1, Supplementary Figure S2), and thus, any of these mutations may influence GDH’s allosteric properties. Of these four mutations, the localization of D495G and A511V close to the Arg496 and Gly509 (originally Arg493 and Gly506 in *Hylobates moloch*) sites suggests they could at least partially resemble features of the R496S and G509A mutations conserved in known GLUD2 from other species (Figure 3, Supplementary Figure S2). The R523H substitution has been studied with the help of mutagenesis of human GLUD1, and no significant effect has been detected when introduced alone [80]. Its role has been supposed as weakening of electrostatic interactions with ADP [39], although one of the main GLUD2 features is its high potency for ADP activation [40,41]. Our revision of the mutation site structure and the binding of NADH within the ADP site suggests that the substitution R523H can reduce electrostatic repulsion between the GDH residue and NADH within the ADP site (Supplementary Figure S2). The mutation M522V takes place in the pivot helix next to the R523H site, but the residue side chain interacts with a NAD-domain helix (Supplementary Figure S2). Residues of this helix are involved in GTP binding. Therefore, the Met522 mutation could influence both GDH activity and responsiveness to GTP inhibition. Thus, of the four D495G, A511V, M522V and R523H mutations, D495G, A511V and M522V could compensate for the reverse mutations S496R and A509G. Anyway, such a hypothesis needs further investigation, as the existence of the GLUD2 variant possessing Arg496 and Gly509 (originally Arg493 and Gly506 in *Hylobates moloch*) significantly questions our understanding of the enzyme function and main differences from GLUD1. Particularly, identification of R496S and G509A as the two positively selected residues was partially based on the fact that all hominoid species preserve these mutations [12]. The GLUD2 variant from *Hylobates moloch* suggests that the R496S mutation, which abolishes the enzyme activity and decreases its stability [45,81], could not only be compensated by an additional G509A mutation [45], but might be eliminated, with alternative mutations compensating for its role. In this regard, sequencing of other hominoid species could provide more relevant details for the understanding of the evolution of human ancestors.

Compared to the mutations accumulated in *Hylobates moloch*, the mutations in the *Symphalangus* and *Nomascus* genera continue the pattern of mutations shared by other branches, which mainly can be characterized as adaptation to already accumulated changes (Table 1). That is, generally, all the mutations of “mature” GLUD2 belong to the following groups. Soon after the *GLUD2* emergence, mutations in proximity to the active (NAD(P)H) site, in proximity to the C-terminal/Leu site and within or close to the antenna occur (Table 1). These mutations, especially R496S and G509A, are known to affect the protein properties and stability, with R496S almost impairing the catalytic function of GDH [45,81]. Further, likely adaptive mutations in all branches include a few mutations within the hydrophobic core of the NAD domain and on its surface. More mutations in proximity to the active (NAD(P)H) site and within/close to the antenna often occur too (Table 1). Many of these mutations occur very close to each other in the structure, independently in different species. A few mutations possess unique novel features such as removal, change or return of a phosphorylatable or acetylatable site (Table 1). In gorilla, the S119C mutation provides the basis for a novel S-S bond formation with C112 from another GDH subunit, which is not observed in other primate GDHs. Mutations within the C-terminal area could influence GLUD2 regulation by its ligands such as leucine and those binding to the bithionol site (Figure 1) [10]. The effect of mutations structurally close to the C-terminus on GDH activation is supported by the data from GDH expression vectors. That is, N-terminal His-tag or FLAG-tag have no effect on GDH regulation by ADP or GTP, while C-terminal FLAG-tag perturbs GDH activation by ADP [92]. No effect of Leu activation has been studied. However, both activators leucine and ADP support each other, likely mediating their action through the K^+^ site [10].

Mutations on the protein surface could affect the participation of GDH in protein–protein interactions. Such interactions may be involved in the formation of filamentous structures by mammalian glutamate dehydrogenases [7,8]. Mammalian GDH also forms a heterologous complex with the mitochondrial transaminase and dehydrogenase of branched-chain 2-oxoacids [93]. Human GLUD1 and GLUD2 differ in their ability to form these complexes and the addition of the transaminase provides protection from GTP inhibition of GDH [93]. A chicken liver GDH has been reported to be a histone H3-specific protease, involved in H3 tail-clipping, which may be a moonlighting function of extramitochondrial GDH [94]. A 50 kDa pig liver protein that corresponds to a membrane-bound isoform of GDH has been identified to have microtubule binding activity [95]. Notably, an integral mitochondrial membrane [2Fe-2S]-containing protein mitoNEET has been discovered to form a disulfide bond between mitoNEET Cys84 residue and a GDH residue corresponding to human Cys376, which increases the GDH catalytic activity *in vitro* [96]. Additionally, mitoNEET can significantly decrease GDH inhibition by palmitoyl-CoA and epigallocatechin gallate [97]. GLUD1 phosphorylated at Ser384 has been shown to interact with nuclear factor RelA and with serine kinase IKKβ in human cancer cells [86]. Both proteins interacting with phosphorylated GLUD1 are involved in the NF-κB signaling pathway. Mutation S384T of GLUD2 can alter such protein–protein interactions of GDH (Table 1). Other mutations of GDH sites involved in posttranslational modifications (Table 1) can also similarly regulate protein–protein interactions of GLUD1 or GLUD2. Participation of GLUD2 in these and other still-unknown protein–protein interactions would likely be affected by substitutions on the protein surface, such as some of the mutations observed in *GLUD2* evolution (Table 1).

Notably, the GLUD2 mutations do not influence the active site, with the exception of regulation of its specificity to NADH or NADPH, and also do not involve residues participating directly in binding of GTP, ADP, leucine or Zn^2+^. Such preservation of the active site and the main regulatory sites of GLUD2 is of interest. In this regard, it may be important to note that cellular localization of human GLUD1 and GLUD2 is mostly identical [98]. To our knowledge, no data on GLUD1/GLUD2 oligomers’ activity have been published; however, there is an example of crystalized oligomeric GDH structure of GdhA and GdhB or GdhB alone in *Thermus thermophilus* ([37]; PDB IDs: 3AOG and 3AOE). The two proteins were co-purified, and the hetero- and homo-oligomeric structures significantly differed in their regulatory properties [99]. Thus, when expressed together, GLUD1 and GLUD2 could form functional hetero-oligomeric complexes, and preservation of the main functions of the enzyme would be necessary for the function of such complexes.

### 3.4. Analysis of Mutation Sites Occuring in the MTS of Ape GDH

The MTS is the sequence of a protein usually localized on its N-terminus that targets the protein to mitochondria. GDH MTS is cleaved after the protein’s import into the mitochondrion, as most often happens with other mitochondrial proteins. The GDH after MTS cleavage is often called “mature”; however, this terminology does not take into account the data on GDH non-mitochondrial localization. For example, potential nuclear localization is suggested from the reported histone H3-specific protease activity of chicken liver GLUD1 [94]. Human GLUD2 immunostaining revealed its co-localization with Lamin A/C on the nuclear membrane in neurons [73]. Cytosolic localization and localization of both human GDHs in the endoplasmic reticulum has been reported [100], which is supported by the potential microtubule binding activity of pig liver GLUD1 [95] and the reported interaction of Ser384-phosphorylated human GLUD1 with nuclear factor RelA in the cytoplasm and with serine kinase IKKβ [86]. Thus, independent data on extramitochondrial localization of both GLUD1 and GLUD2 have been accumulated. Importantly, localization of human GLUD1 and GLUD2 in the cytosol or endoplasmic reticulum is associated with the full-length GDH forms [100].

Still, both human GDHs primarily localize to the mitochondria. The GDH MTS is rather long and is predicted to contain an N-terminal short (approx. ten amino acid residues) α-helix and a second longer one [17]. Removal of the first 15 amino acid residues (the first α-helix) perturbs mitochondrial localization of GDH [17], while the first proposed α-helix (residues 1–10) but not the second α-helix (residues 16–32) of human GLUD2 could alone target a protein to the mitochondria [18]. However, the targeting signal of the first ten GLUD2 amino acid residues alone is weak. Additionally, removal of the first three positive residues of MTS together (mutant R3A-K7A-R13A) perturbed mitochondrial localization, while substitutions of Arg3, Leu5, Lys7 and Arg13 separately or in pairs caused only partial or no effect on mitochondrial transport [18].

The role of positively charged and hydrophobic residues in MTS is well-known. The disruptive effect of total elimination of positively charged residues from the GDH N-terminal sequence on mitochondrial targeting only supports this knowledge. In this regard, it is worth noting that instead of the positive Lys7 residue of GLUD2, negatively charged Glu7 is present in GLUD1 of apes (Figure 3). Moreover, another GLUD1 negatively charged residue, Asp25, is also eliminated independently both in the gibbon branch and in great apes either by a deletion or a substitution for histidine, respectively (Figure 3). Such a difference in the charged residues of GLUD1 and GLUD2 MTS could likely influence the targeting of GLUD2, facilitating its transport to the mitochondria. Still, as mentioned above, extramitochondrial localization of both GLUD1 and GLUD2 is observed. In this regard, data on posttranslational modifications of GDH MTS could improve our understanding of GDH import into mitochondria. It appears that Ser21 of murine GDH can be phosphorylated [101]. Similar phosphorylation of bovine GDH has been observed (our unpublished data). Ser21 is conserved in both GLUD1 and GLUD2; thus, its phosphorylation could affect the localization of both proteins (Figure 3).

With all these data in mind, we can carefully analyze the evolutionary changes in the MTS of GDH. Rosso et al. [19] have proposed that GLUD2 possesses an enhanced mitochondrial targeting specificity. The main role for such an effect is likely due to the mutations of Glu7 and Asp25 soon after the gene duplication. Indeed, the GLUD2 leader sequence fused to GFP exhibited a more efficient mitochondrial targeting compared to the GLUD1 leader sequence [19].

Proline residues disrupt α-helix formation, and thus, one should also pay attention to mutations involving proline residues within the GDH MTS (Figure 3). Of the many substitutions observed in GLUD2, proline has appeared in position 11 of gibbons, gorilla and human (Figure 3). The ancestral state reconstruction analysis shows that the common ancestor of hominoids acquired Pro in the 11th position of GLUD2. Then, reverse mutation to leucine occurred in the orangutan lineage. An additional nucleotide substitution led to a P11T mutation in chimpanzees. When present, Pro11 makes the first α-helix of GLUD2 shorter than that of GLUD1, where Leu11 is encoded, and the first α-helix is limited by the Pro16 residue (Figure 3). Due to the potential effect of phosphorylation of MTS residues, a P41S mutation in the common ancestor of great apes could also be taken into account (Figure 3). Other mutations in the MTS of GLUD2 have no clear pattern.

A few substitutions in GLUD1 after the gene duplication can also be found (Figure 3). The only one of them observed right after the GDH duplication is A23S mutation, which is conserved in all species possessing *GLUD2* gene. Mutation of a hydrophobic Ala for Ser would slightly worsen the mitochondrial transport efficiency. Additionally, since Ser23 is close to the phosphorylatable Ser21, the new Ser23 residue may also be a target of phosphorylation. In the gorilla, chimpanzee and human common ancestor, an R32W mutation occurred, also decreasing the charge of the GLUD1 MTS. However, this residue is localized in the second MTS α-helix, which is less important for targeting than the first one [18]. In the gibbon-branch GLUD1, an R50W mutation is present, which is identical to the presence of Trp50 (originally Trp47) in GLUD2 of *Hylobates moloch* (Figure 3). This position is close to the cleavage site of GDH; therefore, GLUD1’s cleavage efficiency may differ in gibbons and in great apes.

Thus, analysis of GLUD2 and GLUD1 MTS mutations suggests that the efficiency of GLUD2 mitochondrial targeting was improved right after the gene duplication mainly by the elimination of the two negatively charged residues Glu7 and Asp25 from the GLUD2 MTS, while the A23S mutation occurred in the GLUD1 MTS. The novel Ser23 residue is next to the phosphorylatable Ser21 residue, which is conserved in both GDHs and may help GDH to escape from mitochondrial transport through phosphorylation. Such a hypothesis is supported by an association of GDH partial cytosolic localization with its full-length protein form [100]. Thus, although both GDHs can still escape mitochondrial transport, GLUD2 is more prone to mitochondrial targeting, and its existence potentially enabled a reduction in GLUD1 mitochondrial targeting. GLUD2 expression in a rather broad number of cells and tissues and its resistance to GTP inhibition makes this protein helpful for the upregulation of mitochondrial oxidative metabolism. The potential formation of GLUD1-GLUD2 heterohexamers could also regulate GDH’s susceptibility to GTP inhibition.

## 4. Clinically Relevant Genetic Variants of GDH Revealed in Patients

Duplication of the GDH gene is a relatively recent event that took place 18–23 million years ago. Since then, GLUD2 keeps accumulating substitutions, while the GLUD1 protein sequence has been mostly preserved intact, with missense mutations mostly occurring in the MTS. Importantly, some of the GDH variants in the human population are known to be pathogenic, and structural characterization of the available mutations may provide molecular mechanisms for the development of the pathologies.

**Figure 4:**
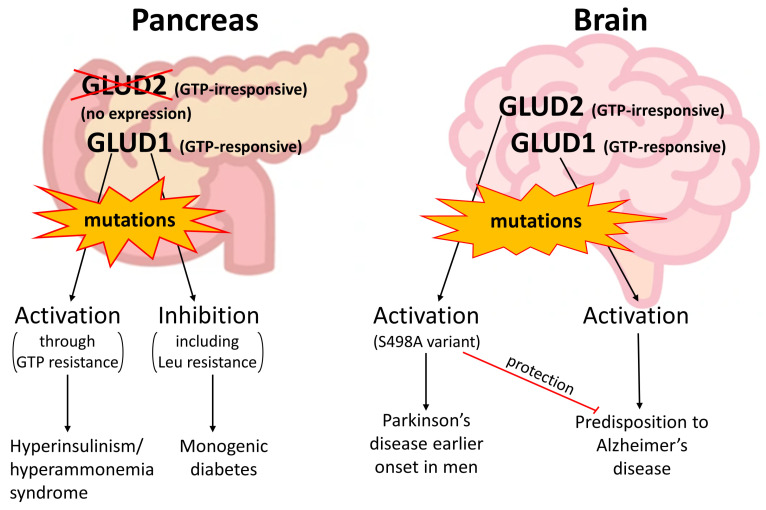
Schematic summary of the consequences of *GLUD2* and *GLUD1* mutations in patients. The two organs involved are the pancreas, where *GLUD2* b-cell expression is normally suppressed, and the brain, where both GDH isoenzymes are expressed in neurons and glial cells. GLUD1 activatory and inhibitory mutations reported in the ClinVar database [109] may cause HI/HA syndrome or monogenic diabetes, respectively. No data on the effect of inhibitory *GLUD1* or *GLUD2* mutations in patients have been reported yet; however, the gain-of-function S498A GLUD2 variant causes predisposition to earlier onset of Parkinson’s disease in men. This GLUD2 mutation also has a protective effect on Alzheimer’s disease, while hyperactivation of GLUD1 may support the predisposition to Alzheimer’s disease in addition to its established role in hyperinsulinemia.

Mutations in the *GLUD1* gene are known to cause hyperinsulinism/hyperammonemia syndrome (HI/HA) (Figure 4, Table 2). These HI/HA-responsible mutations possess a common feature—they disrupt GDH inhibition by GTP [102,103]. As noted, the GTP-resistant *GLUD1* mutations fall into three different categories at the structural level. The mutated residues may (1) form a direct contact with GTP; (2) be localized in the short helix of the descending strand of the antenna, which interferes with the transition between opened and closed conformations of GDH through the pivot helix; or (3) be localized in the ascending strand of the antenna and interfere with intersubunit communication, which is important for GTP inhibition [104]. Thus, the HI/HA-causing mutations of GLUD1 are activatory mutations that block the inhibition of the enzyme by one of its main regulators—GTP (Figure 1, Figure 4). ATP also binds to the GTP site and inhibits GDH. When the molecular mechanism of GTP/ATP inhibition is disrupted, hyperactive GDH fuels the TCA cycle even at high ATP or GTP levels. The latter causes enhanced insulin secretion by the pancreatic b-cells not only upon high blood glucose but also during fasting and after a protein meal, which may enhance GDH activity through activation by leucine [102,105]. Patients with HI/HA often present with generalized epileptic seizures that are resistant to anticonvulsant drugs. The seizures may also occur independently of the hypoglycemic episodes [106]. Some patients may manifest developmental defects, intellectual disability and movement disorders in HI/HA syndrome [107,108]. The syndrome is likely to be manifested at an early age [108].

The resistance of GLUD1 HI/HA-causing variants to GTP makes these mutant protein forms partially similar to GLUD2, which is less prone to GTP inhibition. Then, *GLUD2* expression in b-cells could result in the same enhanced insulin secretion as in HI/HA syndrome caused by *GLUD1* mutations. Such a potentially harmful effect of *GLUD2* expression is prevented by suppressed expression of *GLUD2* in pancreatic endocrine cells, possessing zero expression of *GLUD2* according to the available single-cell data (Figure 4) [55,58]. Thus, downregulation at the expression level is involved in the prevention of possible *GLUD2*-related hyperinsulinism. The mechanism of such downregulation should have emerged right after the gene duplication, since the common ancestor of hominoids already carried the R496S and G509A mutations in the *GLUD2* gene, which are mostly responsible for GTP resistance and stability of the protein (Figure 2 and Figure 3, Table 1).

A study of a mouse model with *GLUD1* overexpression in neurons has observed degeneration of the CA1 hippocampal region, resembling Alzheimer’s disease pathology [71]. Due to the specific promoter, the effect of *GLUD1* activation in neurons could be analyzed without altering the insulin status. It has been suggested that neuronal GDH overexpression accelerates age-related neuronal loss and dendritic dysfunction [71]. These mouse model data indicate that not only HI/HA syndrome but also age-related disorders such as Alzheimer’s disease could be associated with the *GLUD1* mutations. The association of hyperinsulinemia with the risk of Alsheimer’s disease has also been reported in patients (Figure 4) [110]. The neuron-specific GDH hyperactivation by genetic *GLUD1* overexpression in mice is partially similar to the presence of GTP-resistant *GLUD2* in humans. As noted above, *GLUD2* expression in excitatory neurons is relatively high, although lower than in Sertoli cells or cytotrophoblasts. In contrast, brain inhibitory neurons possess approximately three times lower *GLUD2* expression compared to excitatory neurons [55,58]. As a result, hyperactivation of excitatory neurons due to the presence of GTP-insensitive *GLUD2* may occur. Indeed, Alzheimer’s disease is characterized by cortical and hippocampal hyperactivity in the early stages of disease, progressing to hypoactivity during later stages of neurodegeneration [111].

In the *GLUD2* gene, a relatively frequent (Table 2) polymorphism resulting in an S498A substitution is associated with Parkinson’s disease onset (Figure 4) [112]. The link between the earlier onset of Parkinson’s disease and the S498A mutation was found in men but not in women, which can be explained by the location of the *GLUD2* gene on the X chromosome [28]. The significance of such a *GLUD2* mutation has been shown in a mouse model. A decrease in glutamate transport into glial cells and an increase in glutamate neurotoxicity, leading to the death of neurons in the substantia nigra, has been reported [113]. This was accompanied by a decreased level of succinate dehydrogenase and downregulation of oxidative metabolism in glial cells. However, the S498A *GLUD2* variant was less frequent (2.11%) in Alzheimer’s disease patients compared to controls (16%; *p* < 0.01) [114]. Therefore, a protective effect of the S498A mutation from Alzheimer’s disease is suggested (Figure 4) [114], although the mutation itself is associated with the earlier onset of Parkinson’s disease [112].

The currently available polymorphisms of the GDH genes resulting in amino acid substitutions are summarized in Table 2. HI/HA syndrome is the most prominent known manifestation among the GDH mutations (Table 2). It is developed due to the hyperactivation of the oxidative metabolism in pancreatic b-cells, where no expression of *GLUD2* is normally observed. Other pathologic conditions may also be caused by *GLUD1* mutations, but such events are rare (Table 2). Neuronal or glial expression of *GLUD1* or *GLUD2*, respectively, can lead to age-related disorders [28,71,112], but the link between these polygenic pathologies and the mutations in GDH genes are more difficult to establish. It can be suggested that GDH mutations are partially responsible for the development of age-related neurodegeneration, and thus, more efforts are required for genetic studies of these disorders.

**Table 2:** Missense variants of GDH (*GLUD1* or *GLUD2* genes) observed in patients. Data from the ClinVar database [109] are used. Only variants reported by two or more submitters are considered. The medical condition is written as submitted to ClinVar. Structural position of the mutations is provided as assessed in this work. HI/HA—hyperinsulinism/hyperammonemia syndrome; FH—familial hyperinsulinemia; MD—monogenic diabetes; PD—Parkinson’s disease, late-onset.

Variant	Allele Frequency	Submitted Condition	Structural Data	References
*GLUD1*				
A18V	–	HI/HA	Modified MTS sequence	–
G35E	–	HI/HA; MD	Modified MTS sequence	–
Q36R	0.00280	HI/HA	Modified MTS sequence	–
H52N	0.00002	HI/HA	Modified MTS sequence	–
D126N	0.00839	HI/HA; MD	Mutation at the entrance to the Leu site, hexachlorophene and GW5074/bithionol sites. May affect regulation by Leu.	–
R274C	–	HI/HA	A GTP-binding residue. Decreases GTP inhibition.	[115,116,117,118,119]
R322H	–	HI/HA	A GTP-binding residue. Decreases GTP inhibition.	[115,116,117]
S357F	–	HI/HA	A phosphorylatable [120] residue at the entrance to the NADPH site.	–
D375N	0.00003	Uncertain	Mutation at the surface of the NAD domain.	–
P489R	–	HI/HA	A residue between two helices of the antenna. Should affect allosteric regulation of GDH.	–
S498L	–	HI/HA	A residue of the antenna, small helix. Should affect allosteric regulation of GDH (see S498A below).	[102,107,116,118,121,122,123,124]
H507Y	–	HI/HA; FH	A GTP-binding residue. Decreases GTP inhibition.	[102,125]
R523H	–	HI/HA	A pivot helix residue, substituted in *GLUD2*. May slightly affect ADP activation [39].	–
N551S	–	HI/HA	A residue in proximity to the C-terminal/Leu site, substituted in *GLUD2*. May influence Leu activation and hexachlorophene and inhibition by GW5074 or bithionol.	–
*GLUD2*				
S498A	0.03285	PD	A residue of the antenna, small helix. Activity and allosteric regulation by estrogens are affected [112].	[112]

Of note, the S498A *GLUD2* variant is the most frequently observed GDH mutation (Table 2). It is about four times more frequent than the D126N of *GLUD1*. All the other *GLUD1* variants with established allele frequency, namely, Q36R, H52N and D375N, are 10–1000 times less frequent compared to S498A of *GLUD2*. Importantly, no allele frequency is available in the ClinVar database for the variants known to be responsible for HI/HA with strong clinical symptoms (Table 2), suggesting these variants are even less frequent. In other words, GDH mutations with early pathogenic manifestation such as HI/HA syndrome are much rarer compared to the mutations associated with age-related disorders such as Parkinson’s disease with late onset (Table 2).

Of the available *GLUD1* mutations with calculated frequencies, D126N is the most frequent one (Table 2). Unlike the reported HI/HA-causing mutations associated with disrupted GTP inhibition [104], the D126N substitution likely affects GDH activation by leucine and/or inhibition by hexachlorophene, GW5074 or bithionol (Table 2, Figure 1). Thus, D126N is likely not involved in the mechanism of HI/HA syndrome caused by the disruption of GDH inhibition by GTP/ATP in pancreatic b-cells. Instead, this mutation may influence GDH activation by leucine. The Q36R and H52N mutations are localized in the MTS of GDH. Substitutions within this region can influence GDH mitochondrial targeting, although individual substitutions do not affect it dramatically [18]. From the available allele frequencies, one can suggest that the H52N mutation is more pathogenic (Table 2). Such an effect may be due to the potential influence on the MTS peptide cleavage, which site is located next to the mutated position. The D375N mutation is localized on the surface of the NAD domain, and its pathogenicity is not established. The mutation is rare (0.00003 allele frequency), but no clear effect on GDH regulation can be proposed from the structural data.

Thus, from the reported clinical data, it can be concluded that mutations in the GDH genes cause either (1) HI/HA syndrome, which develops pathological effects at an early age, and which is caused by very rare *GLUD1* mutations resulting in GDH resistance to inhibition by GTP; (2) Parkinson’s disease with late onset, which is caused by a rather frequent (0.03) variant of *GLUD2*, resulting in decreased activity and altered inhibition of this enzyme by estrogens; (3) other conditions, where HI/HA syndrome may be suspected due to the mutations found in the *GLUD1* gene, but the mutations are not so rare (up to 0.008) and are not associated with disrupted GTP inhibition of GDH. Available mutations of GDH with uncertain pathogenicity and a potential effect of both *GLUD1* and *GLUD2* on the development of age-related neurological disorders, including Alzheimer’s and Parkinson’s disease [71,112], provide valuable material for further evaluation of the clinical role of both GDH genes in patients.

## 5. Conclusions

This contemporary review of sequencing data from primates allowed us to revisit the evolution of the *GLUD1* and *GLUD2* genes. New *GLUD2* sequences from gibbons provide an example of *GLUD2* with reverse mutations to those suggested as the main ones responsible for the lower sensitivity of GLUD2 to GTP. Instead, alternative mutations are accumulated in this GLUD2 variant, potentially also affecting the sensitivity to GTP inhibition. Use of the latest structural data on GDH ligands, including a recently identified leucine activatory site, provides a broader understanding of the consequences of *GLUD2* mutations for its regulation by the ligands. Known GDH posttranslational modifications and mutations of their sites are considered. Together with the data on protein–protein interactions and subcellular localization, these data elucidate a potential difference in GLUD1 and GLUD2 involvement in signaling pathways in the cytoplasm/nucleus and formation of supramolecular complexes involved in oxidative metabolism in the mitochondria. Analysis of mutations in the MTS reveals not only mutations enhancing mitochondrial targeting of GLUD2 right after the gene duplication, but also a GLUD1 mutation likely reducing its mitochondrial targeting. Novel structural data are applied for a summary of GDH variants available in patients. Structural/functional relationships and the potential effect of known GDH mutations on hyperinsulinism/hyperammonemia syndrome, Parkinson’s, and Alzheimer’s diseases are reviewed. These results provide novel understanding and raise new questions about the evolution and regulation of an enzyme possessing several functions in the cell and indispensable for the central metabolism of all tissues. Our review elucidates the lack of understanding of *GLUD2* evolution, which may be responsible for the development of the human brain. In this regard, more efforts are required for the sequencing of rare and extinct hominoid species. More attention should be paid to the genetic variants of *GLUD1* and *GLUD2* in patients with hyperinsulinism, inherited predisposition to diabetes, as well as Parkinson’s and Alzheimer’s diseases.

## Figures and Tables

**Table 1:** Amino acid substitutions accumulated in the “mature” GLUD2 sequences after the gene duplication. The substitutions are provided in the evolutionary order according to Figure 2, with corresponding clades (A–I) listed in the first column. Residue numbers are provided according to the common ancestor or human sequence (Figure 3). Structural localization in the chemically significant or regulatory sites is considered. Repeated positions of mutations are marked with letters in superscript (a–f).

Clade (Figure 2)	Group/Organism	Substitution	Structural Area	Unique Novel Feature	References
A	Hominoidea	A56V ^a^	Proximity to NAD(P)(H) site	–	
	Hominoidea	S227N	Proximity to NAD(P)(H) site	Phospho-Ser site removal	[78,79]
	Hominoidea	E87K	Proximity to C-terminal site	–	
	Hominoidea	D195E	Proximity to C-terminal site	–	
	Hominoidea	N551S	Proximity to C-terminal/Leu site	–	[80]
	Hominoidea	R496S ^b^	Within/close to antenna	Altered allosteric regulation	[45,81,82,83]
	Hominoidea	G509A ^c^	Within/close to antenna	Altered stability	[45,80,82]
B	Hylobatidae	T154A ^#^	Proximity to NAD(P)(H) site	Phospho-Tre site removal	[78]
	Hylobatidae	L430V ^#^	Hydrophobic core of NAD domain	–	
	*Hylobates*	E61K ^#^	Proximity to NAD(P)(H) site	–	
	*Hylobates moloch*	D495G	Within/close to antenna	Altered regulation (predicted)	This work
	*Hylobates moloch*	S496R ^b^	Within/close to antenna	Reversed mutation	
	*Hylobates moloch*	A509G ^c^	Within/close to antenna	Reversed mutation	
	*Hylobates moloch*	A511V	Within/close to antenna	Altered regulation (predicted)	This work
	*Hylobates moloch*	M522V	Close to antenna/Hydrophobic core of NAD domain	Altered regulation/activity (predicted)	This work
	*Hylobates moloch*	R523H ^d,^**	Proximity to ADP site	–	[80]
	*Symphalangus*	S140R	K^+^ site	Altered Leu-K-ADP interaction	[10]
	*Symphalangus*	N196K	Proximity to C-terminal site	–	
	*Symphalangus*	Y289H	Hydrophobic core/surface of NAD domain	–	
	*Symphalangus*	N406S	NAD(P)(H) site	–	
	*Nomascus*	A137T	Protein hydrophobic core/surface	–	
	*Nomascus*	S336N	Surface of NAD domain	Phospho-Ser site removal	[79]
	*Nomascus*	H481R	Within/close to antenna	–	
C	Hominidae	V56L ^a^	Proximity to NAD(P)(H) site	–	
	Hominidae	S384T	Proximity to NAD(P)(H) site	Phospho-Ser/Tre site modification	[79,84,85,86]
	Hominidae	K352R ^e^	Proximity to NAD(P)(H) site	Ac-Lys site removal	[87,88,89]
	Hominidae	R92Q	Proximity to C-terminal site	–	
	Hominidae	M423L	Hydrophobic core of NAD domain	–	[81]
	Hominidae	R523H ^d,^**	Proximity to ADP site	–	[80]
D	*Pongo*	I292N	Surface of NAD domain	–	
	*Pongo*	L293V	Hydrophobic core of NAD domain	–	
	*Pongo*	I328V	Hydrophobic core of NAD domain	–	
	*Pongo*	L428V	Hydrophobic core of NAD domain	–	
	*Pongo*	Q494R	Within/close to antenna	–	[90]
E	Homininae	A374V ^f^	Hydrophobic core of NAD domain	–	
	Homininae	I219V	Hydrophobic core of NAD domain/hexachlorophene site	–	
	Homininae	G300R	Surface of NAD domain	–	
F	*Gorilla*	S119C	Subunit interface	Novel S-S bond formation	
	*Gorilla*	K415R	Surface of NAD domain	Ac-Lys site removal	[88,89]
	*Gorilla*	L418Q	Surface of NAD domain	–	
	*Gorilla*	E492D	Within/close to antenna	–	
G	Hominini	–	–	–	
H	*Pan*	I358L	Hydrophobic core of NAD domain	–	
	*Pan*	V374I ^f^	Hydrophobic core of NAD domain	–	
I	*Homo*	R352K ^e^	Proximity to NAD(P)(H) site	Ac-Lys site return	[87,88,89]
	*Homo*	M468L	Within/close to antenna	–	[81]

^#^—also detected in *Hylobates lar* [12]; **—independent identical mutations in Hominidae and *Hylobates moloch* according to ancestral sequence reconstruction using maximum likelihood method [74] under the Kimura 2-parameter model [75] implemented in MEGA11 [76].

## Data Availability

The presented data are available in this article and Supplementary Materials.

## Supplementary Materials

The following supporting information can be downloaded at: https://www.mdpi.com/article/10.3390/ijms25084341/s1.

## Abbreviations

GDH	glutamate dehydrogenase,
MTS	mitochondrial targeting sequence,
HI/HA	hyperinsulinism/hyperammonemia syndrome.
