# Supplementary material for: Evolutionary Changes in Primate Glutamate Dehydrogenases 1 and 2 Influence the Protein Regulation by Ligands, Targeting and Posttranslational Modifications
Source: Int J Mol Sci. 2024 Apr 14;25(8):4341. doi: 10.3390/ijms25084341 (PMC11050691; doi:10.3390/ijms25084341)
Supplement: Supplementary file 1 [file ijms-25-04341-s001.zip › ijms-2942836-supplementary.pdf]

International Journal of *Molecular Sciences*

Evolutionary Changes in Primate Glutamate Dehydrogenases 1 and 2 Influence the Protein Regulation by Ligands, Targeting & Posttranslational Modifications

Yulia A. Aleshina [1,2] and Vasily A. Aleshin [3,4,*]

1 Martsinovsky Institute of Medical Parasitology, Tropical and Vector Borne Diseases, Sechenov First Moscow State Medical University, 119435 Moscow, Russia; vjulia94@gmail.com.
2 Faculty of Bioengineering and Bioinformatics, Lomonosov Moscow State University, 119234 Moscow, Russia.
3 Belozersky Institute of Physicochemical Biology, Lomonosov Moscow State University, 119234 Moscow, Russia; aleshin_vasily@mail.ru
4 Department of Biochemistry, Sechenov First Moscow State Medical University, 119048 Moscow, Russia.
* Correspondence: aleshin_vasily@mail.ru;

Supplementary material

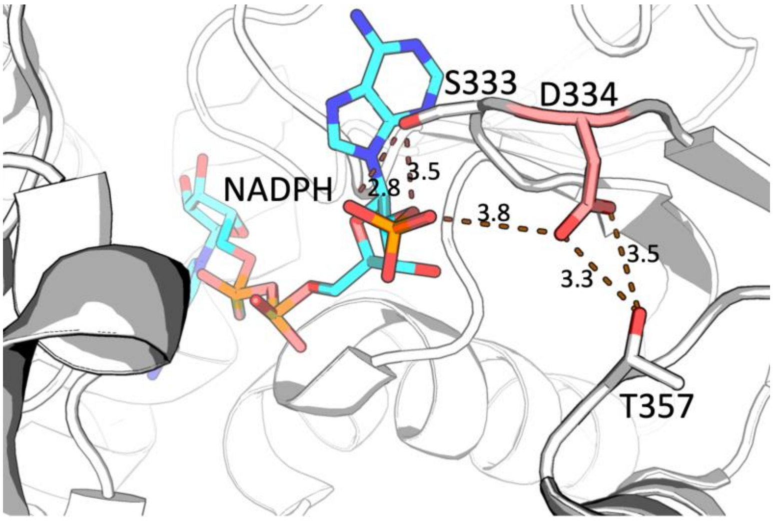

Supplementary Figure S1. Localization of the D334 residue within a GDH structure with NADPH (PDB ID: 3ETE, chain A). Residues numbering is provided in accordance with the human sequence and the numeration used throughout the paper (Fig. 3). The D334 residue (D277 of the PDB model) is colored in pink, NADPH is colored in cyan. The S333 and T357 residues (S276 and T300 of the model, respectively), as well as the cartoon model of GDH, are shown in light gray. Non-carbon atoms are shown using a standard color code. Distances (Å) between atoms are shown with brown dashed lines.

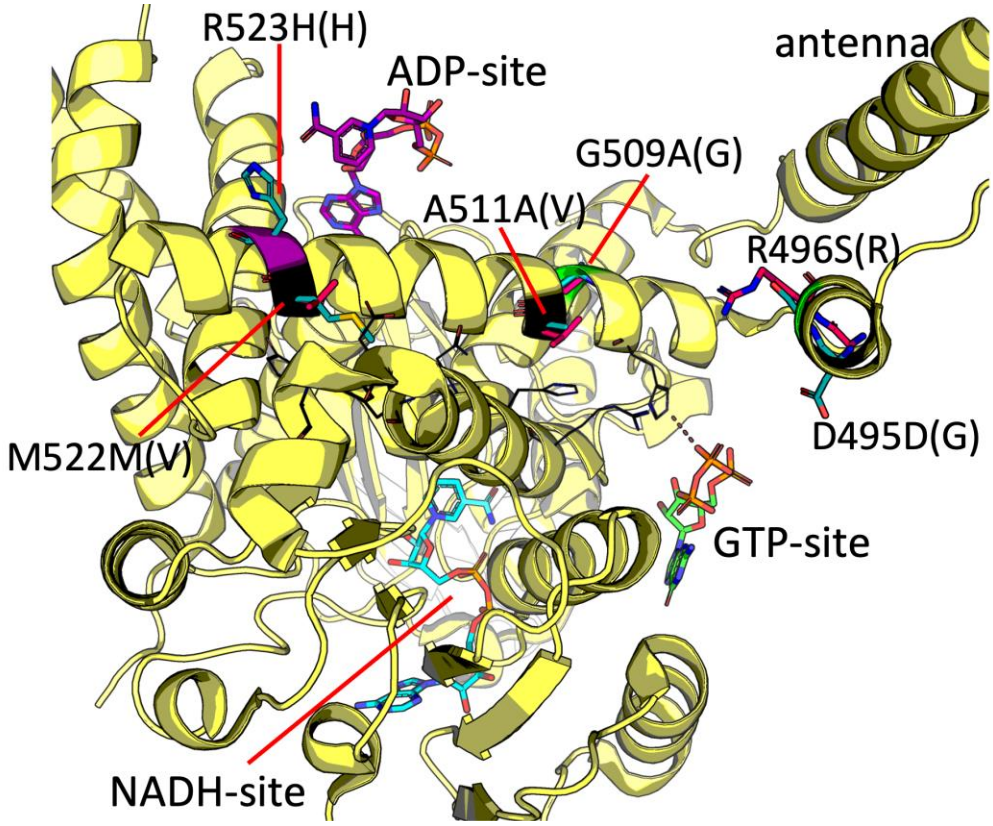

Supplementary Figure S2. A close-up view of a GLUD2 subunit from the antenna. The chain A of the only available GLUD2 structure (PDB ID: 6G2U) from human is used as the protein model. The ligands are added with the help of a structural alignment with the GLUD1 structure (PDB ID: 3JD3) possessing NADH in the active site (carbons are shown in cyan), GTP (shown in green) and NADH in the ADP-site (shown in purple). The mutations sites found in the antenna region and pivot helix of *Hylobates moloch* are indicated in the figure. Residues numbering is provided according to the full-length ancestor of primate GLUD2 or human GLUD2 sequence as used throughout the paper. For each residue, first the ancestral (Fig. 3) residue name is written using standard one-letter code followed by the residue number. Then the residue name of the human GLUD2 is written and the residue name in the *Hylobates moloch* is provided in brackets. The original residue numbers of *Hylobates moloch* are three residues less (e.g. R493 instead of R496) due to a deletion in MTS (Fig. 3). The two mutation sites, R496S and G509A, with reverse mutations in *Hylobates moloch*, are indicated with green cartoon. The mutation site R523H, obtained independently in the two evolutionary branches (*Hylobates moloch* and *Hominidae*), is indicated with purple cartoon. The three sites with novel mutations observed within the antenna or pivot helix in *Hylobates moloch* (D495G, A511V, and M522V) are indicated with black cartoon. Mutated variants of the *Hylobates moloch*-specific residues are prepared using PyMol. Carbon atoms of residues in these three sites are colored in teal or pink for the human or the gibbon variants, respectively. Residues surrounding M522 and A511 side chains are indicated with black lines in order to show better the possible effect of these mutations. Non-carbon atoms are shown using a standard color code.
